# Facile Conversion of the Quinone-Semicarbazone Chromophore of Naftazone into a Fluorescent Quinol-Semicarbazide: Kinetic Study and Analysis of Naftazone in Pharmaceuticals and Human Serum

**DOI:** 10.3390/s22166205

**Published:** 2022-08-18

**Authors:** Mohammed Gamal, Hazim M. Ali, Rania El-Shaheny, Ibrahim A. Naguib, Izzeddin Alsalahat, Mahmoud El-Maghrabey

**Affiliations:** 1 Department of Pharmaceutical Chemistry, College of Pharmacy, Jouf University, P.O. Box 2014, Sakaka 72388, Aljouf, Saudi Arabia or; 2 Department of Pharmaceutical Analytical Chemistry, Faculty of Pharmacy, Beni-Suef University, Alshaheed Shehata Ahmed Hegazy St., Beni-Suef 62574, Egypt; 3 Department of Chemistry, College of Science, Jouf University, P.O. Box 2014, Sakaka 72388, Aljouf, Saudi Arabia; 4 Department of Pharmaceutical Analytical Chemistry, Faculty of Pharmacy, Mansoura University, Mansoura 35516, Egypt; 5 Department of Pharmaceutical Chemistry, College of Pharmacy, Taif University, P.O. Box 11099, Taif 21944, Mecca, Saudi Arabia; 6 UK Dementia Research Institute Cardiff, School of Medicine, Cardiff University, Cardiff CF24 1TP, UK

**Keywords:** naftazone, potassium borohydride, fluorimetry, serum samples, reaction kinetics, green chemistry

## Abstract

Naftazone is a quinone-semi carbazone drug that possesses a strong orange color, and hence it was usually analyzed colorimetrically or by HPLC-UV. However, these methods are not sensitive enough to determine naftazone in biological samples. Naftazone lacks intrinsic fluorescence and does not possess easily derivatizable functional groups. In this contribution, we introduced the first spectrofluorimetric method for naftazone assay through reduction-elicited fluorogenic derivatization through the reduction of its quinone-semicarbazone moiety to the corresponding quinol-semicarbazide derivative by potassium borohydride as a reduction probe. The solvent-dependent fluorescence of the reaction product was studied in various protic and aprotic solvents. Eventually, the fluorescence of the reduced naftazone was measured in 2-propanol at λ_emission_ of 350 nm after excitation at λ_ecxitation_ of 295 nm. The relative fluorescence intensity was linearly correlated to the drug concentration (r = 0.9995) from 10.0 to 500 ng/mL with high sensitivity, where the lower detection limit was 2.9 ng/mL. Hence, the method was effectively applied for naftazone tablets quality control with a mean %recovery of 100.3 ± 1.5, and the results agreed with those of the comparison HPLC-UV method. Furthermore, a new salting-out assisted liquid-liquid extraction (SALLE) method was established for naftazone extraction from human serum, followed by its determination using the developed reduction-based fluorogenic method. The developed SALLE method showed excellent recovery for naftazone from human serum (92.3–106.5%) with good precision (RSD ≤ 6.8%). Additionally, the reaction of naftazone with potassium borohydride was kinetically monitored, and it was found to follow pseudo-first-order kinetics with an activation energy of 43.8 kcal/mol. The developed method’s greenness was approved using three green analytical chemistry metrics.

## 1. Introduction

Naftazone (NFZ, 1,2-naphthoquinone 2-semicarbazone) is a hemostatic and veinotonic agent used for treating diabetic retinopathy and lower limbs venous insufficiency [1]. Furthermore, it has been proved to have good efficacy in patients with parkinsonism and dyskinesia, which attracts attention to it as a promising future non-dopaminergic treatment of these diseases [2]. NFZ is given orally in a total daily dose of 30 mg [1]. The analytical literature cited a few methods for the determination of NFZ, including spectrophotometry owing to its strong orange color [3], voltammetry [4,5,6], thin layer chromatography [7], HPLC [8], and LC-MS/MS [9]. These methods entail some disadvantages; for example, the voltammetry [4,5,6] and thin layer chromatography [7] methods for NFZ are not well-suited for quality control laboratories due to poor sample throughput. At the same time, the spectrophotometric methods [3] are not adequately sensitive. On the other hand, the LC-MS/MS [9] needs a highly skilled operator and expensive instrument (EUR 100,000) that may be of limited availability in many quality control laboratories.

**Figure 1:**
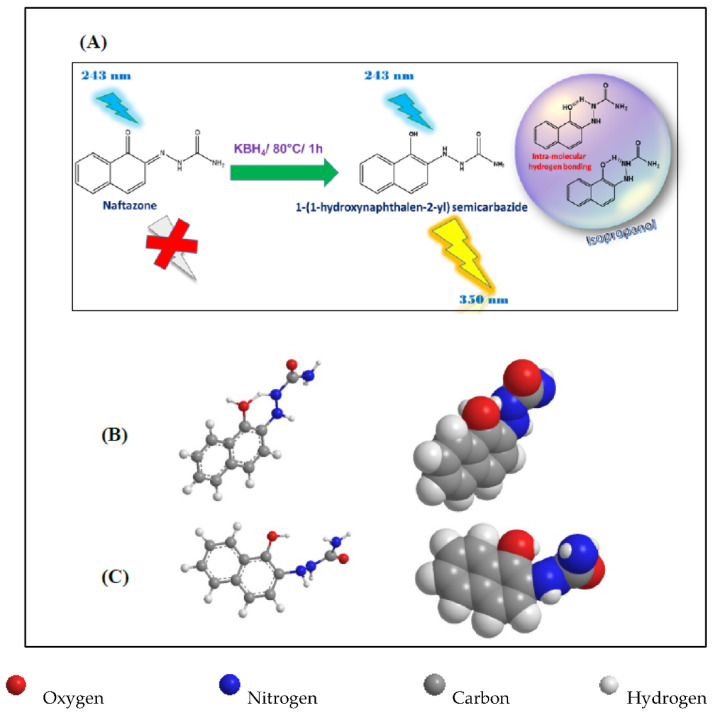
(**A**) Schematic illustration of the principle of the fluorimetric determination of NFZ via reduction with potassium borohydride and the intramolecular H-bonding of NFZ in 2-propanol. Molecular structures (ball and stick on the left and space-filling on the right) for (**B**) intramolecular hydrogen-bonded reduced NFZ and (**C**) reduced NFZ showing their geometric configurations. The structures were generated using Chem3D Ultra 8.0 (Cambridge soft Corporation, Cambridge, MA, USA). The energy minimization function was used to obtain the space arrangement with net inter-atomic forces close to zero, and the position on the potential energy surface is a stationary point.

Despite the fact that fluorimetric analysis is generally recognized for its prominent advantages over other analytical techniques, such as high sensitivity, great specificity, and simplicity, no fluorimetric assay was reported for the determination of NFZ. Thus, we initially investigated the intrinsic fluorescence of NFZ to realize the chance of direct fluorescence measurement without derivatization steps. Nevertheless, it was evidenced that it is non-fluorescent in different inspected solvents. This is because strong-colored drugs that contain quinone adducts have difficulty forming fluorescent products due to their strong absorbance, which might cause self-quenching due to the inner filter effect [10]. Furthermore, it was challenging to develop a fluorescence derivatization reaction for NFZ since it lacks functional groups (Figure 1) suitable for derivatization by common derivatizing reagents such as dansyl chloride [11], fluorescamine [12], NBD-Cl [13], and *o*-phthalaldehyde [14] that are common derivatizing agents for amines, and dinitrophenyl hydrazine that is used for derivatization of reactive carbonyl functionality [15]. A close examination of NFZ structure showed that it is a semicarbazone of 1,2-naphthoquinone, which encouraged the authors to investigate its reaction with a reducing agent to generate a fluorophore through reduction of the quinone-semicarbazone moiety (Figure 1).

Experimental studies showed that the reduction of NFZ with potassium borohydride resulted in the production of a reaction product with intense fluorescence. Hence, we conducted the current study to investigate the kinetics and mechanism of this reaction and to design, validate, and apply the first spectrofluorimetric method for the determination of NFZ in pharmaceutical preparations with high sensitivity. Additionally, the reaction kinetics and activation energy were investigated. Moreover, we evaluated the ecological and safety impacts of the developed method as a foremost step for environmental conservation [16].

## 2. Experimental

### 2.1. Apparatus

A Shimadzu spectrofluorimeter (model RF-1501) (Kyoto, Japan) with a Xenon arc lamp was used for fluorescence measurements with a 1 cm quartz cell. The emission slit width was adjusted to 10 nm. The pH was measured with a pH meter from Hanna Instruments, Inc., Carrollton, TX, USA.

### 2.2. Materials and Solutions

An authentic sample of NFZ (99.9% purity as certified) (Batch No. 0301030075) was presented by Alkan Pharma Co., City of 6th of October, Egypt. Potassium borohydride, methanol, and 2-propanol were bought from ADWIC Co. (Cairo, Egypt). Mediaven tablets containing 5 mg NFZ/tablet produced by Alkan Pharma Co., was purchased from a local pharmacy. A 2.5 mM solution of potassium borohydride was prepared in methanol. NFZ standard methanolic solution containing 100 µg/mL was prepared and proper dilution was completed using methanol to prepare the working solution (10 µg/mL).

### 2.3. Procedure for the Calibration Curve

Different volumes of NFZ standard solution were mixed with 2.0 mL of 2.5 mM potassium borohydride and heated at 80 °C in a thermostatically controlled water bath for 60 min. Solutions were made up to 10 mL in calibrated volumetric flasks using 2-propanol as a diluting solvent; then, they were well mixed. The fluorescence of these solutions was determined at an emission wavelength of 350 nm after excitation at 295 nm. Reagent blank was prepared simultaneously and scanned similarly at these excitation/emission wavelengths. The relative fluorescence intensities (RFIs) were calculated as:

**RFI = fluorescence of the sample − fluorescence of the blank** (1)

Each experiment was repeated three times, and the mean RFIs values were graphed versus drug concentration (ng/mL) to generate the calibration curve, and the regression equation was calculated.

### 2.4. Procedure for Determination of NFZ in Tablets

The weight of 10 tablets was measured, then the tablets were triturated and well mixed. An accurately weighed amount of the powder containing 10 mg of NFZ was transferred to a 100 mL volumetric flask and dissolved in methanol with the aid of ultrasonication for 30 min. The volume was adjusted to 100 mL with the same solvent, followed by filtration to remove insoluble additives and excipients. Different volumes of the filtrate were transferred to 10 mL volumetric flasks, and the procedure for the calibration curve was performed.

### 2.5. Extraction of Naftazone from Human Serum Samples Using Salting-Out Liquid–Liquid Extraction (SALLE)

An aliquot of 300 μL of human serum was mixed with 600 μL acetonitrile, then vortex mixed for one minute. Next, 0.2 g of NaCl was added, followed by centrifugation for 10 min at 2500× *g*. Then, 400 μL of the salted-out acetonitrile upper phase was transferred to a 2.0 mL Eppendorf tube. Then, 400 μL of 2.5 mM potassium borohydride was added, followed by heating at 80 °C in a thermostatically controlled water bath for 60 min. Solutions were made up to 2.0 mL using 2-propanol as a diluting solvent; then, they were well mixed. The fluorescence of these solutions was determined at an emission wavelength of 350 nm after excitation at 295 nm.

### 2.6. Validation Procedure for the Determination of Naftazone in Human Serum

The validation of the developed method for bioanalysis of naftazone in human serum was carried out following the FDA bioanalytical method validation guidance [17]. Calibration curves, the limit of quantification (LOQ), accuracy, precision, and recovery were performed in accordance with FDA guidelines.

The calibration curve for naftazone in human serum was constructed by spiking serum samples with six different concentration levels of naftazone starting from the LOQ and plotting the RFI *versus* concentration (ng/mL). The LOQ was defined as the smallest concentration used in the calibration that showed enough accuracy (80–120%) and precision (RSD of ≤20%) [17]. Moreover, the limit of detection (LOD) was estimated at a ratio of signal-to-noise (S/N) = 3. To study the accuracy and precision of the method, replicate analyses of serum samples spiked with the analytes at LOQ (10 ng/mL) and different concentrations levels (50, 100, 200, 500 ng/mL) were carried out. The average of three measurements for every concentration was calculated. The accuracy was expressed as the difference between the average from the true value. The precisions were calculated as the %RSD for three measurements for every concentration [17].

Furthermore, the absolute recoveries of the SALLE method were evaluated at the LOQ (10 ng/mL) and different concentration levels (50, 100, 200, 500 ng/mL). It was calculated according to the following equation:

%Recovery = (RFI of naftazone extracted from serum samples/RFI of unextracted standard solution) × 100

### 2.7. Procedure for Determination of the Order of the Reaction

Different concentrations of NFZ (50, 100, 200, 400, and 500 ng/mL) were allowed to react with a fixed concentration of potassium borohydride (2.5 mM) for increasing time intervals under the optimum reaction conditions. Plots of product concentration (ng/mL) versus time (seconds) were constructed. The slopes of the obtained curves express the initial reaction rate (**K**) for each concentration. Then, log **K** was plotted versus log **[NFZ]** according to the following equation [18]:

**Log K = Log K’ + n Log [NFZ]** (2)

where **K**: initial reaction rate, **K’**: rate constant, **[NFZ]** is the molar concentration of NFZ, and n: order of the reaction. The order of the reaction was determined using the calculation of n from the slope of the obtained straight line.

### 2.8. Procedure for Calculation of the Rate Constant, Arrhenius Equation, and Activation Energy of the Reaction

The reaction of a fixed concentration of NFZ (1.9 μM) and potassium borohydride (2.5 mM) was carried out at various temperature settings ranged from 50 to 80 °C. Samples were taken and analyzed by the proposed method at different time intervals. Plots of ln (a/(a−x)) versus time t (seconds) were constructed at each temperature according to the following equation [18]:

**ln (a/a−x) = K’ t** (3)

where a is the initial drug concentration (ng/mL), a−x is the remaining drug concentration (ng/mL), t is the time (seconds), and K’ is the rate constant.

The rate constants K’ at each temperature were calculated from the slopes of the obtained curves. Then, Ln K’ was plotted versus 1/T to construct the Arrhenius plot according to Equation (4) [18]:

**Ln K’ = log A − E_a_/RT** (4)

where E_a:_ the activation energy (kcal/mol), R: gas constant = 1.987 kcal.mol^−1^K^−1^, A: frequency factor, and T: temperature (K). The activation energy of the reaction was calculated from the slope of the obtained straight line (slope = −E_a_/R).

## 3. Results and Discussion

### 3.1. The Idea of the Fluorogenic Assay Reaction and Fluorescence Features

NFZ intact drug was found to be non-fluorescent in different investigated solvents. Considering the chemical structure of NFZ would help understand this behavior. From this standpoint, NFZ showed a substituted 1,2-naphthoquinone-semicarbazone moiety (Figure 1A). The carbonyl functionality at 1-position results in the mixing of spin and orbital electronic motion of the aromatic moiety. The spin-orbital coupling abolishes the molecular spin and stimulates intersystem crossing to the triplet state from the singlet one. Consequently, the lowest triplet state is populated at the cost of the lowest excited singlet state, and the energy is lost via non-radiative transition between electronic states [19]. In addition, naftazone is strongly colored [3], hence it suffers from the self-inner-filter effect. Owing to the previously mentioned reasons, NFZ molecules are not fluorescent at all.

In turn, herein, we suggested the possibility of reduction-activated fluorogenic derivatization of NFZ through the reduction of the quinone-semicarbazone moiety to quinol-semicarbazide moiety [20,21] to produce an intensely fluorescent product (Figure 1A). The high fluorescence of the reduced NFZ is attributable to extending the conjugated system and converting the electron-withdrawing carbonyl group into an electron-donating hydroxyl group (Figure 1A). The role of the carbonyl group in spin-orbit mixing is now eliminated. Instead, the hydroxyl group enhanced the fluorescence by donating electrons to the aromatic ring [19]. In addition, the potassium borohydride reduction of the quinonic compound naftazone leads to its decolorization [22] due to its change from the quinone-semicarbazone form to the quinol-semicarbazide potassium borohydride, and hence suppress the self-inner-filter quenching effect. Furthermore, it seems that intramolecular hydrogen bonding of the phenolic O-H and the nitrogen of the hydrazine side-chain resulted in the formation of a six-membered ring which assists the co-planarity of the molecules and, in turn, favors the fluorescence (Figure 1A) [23].

In this study, potassium borohydride was the selected reducing agent since it is effective for the reduction of the carbonyl group of aldehydes and ketones to the hydroxyl group. Additionally, it cannot reduce esters, acids, amides, carbonates, carbamates, and many other groups unless some special catalysts or solvents are added or harsher conditions are applied [24,25,26]. As well, its handiness, low cost, considerable stability, simplicity of work-up, and high product yield make it a widely used reducing agent for aldehydes and ketones [27].

**Figure 2:**
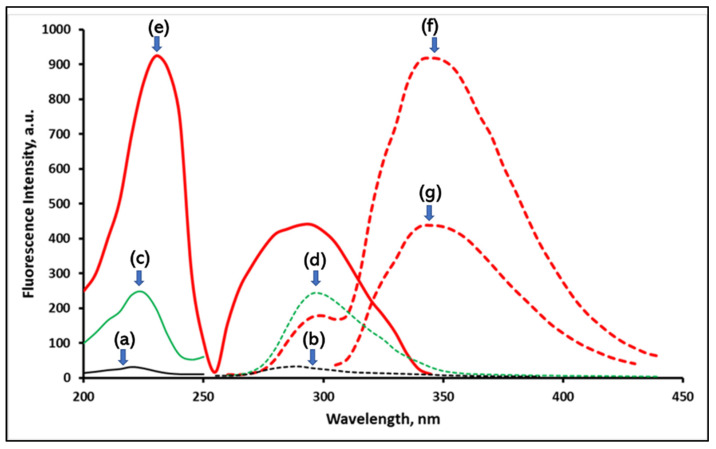
Excitation and emission spectra of NFZ alone (a, b), blank reducing agent (c, d), and reduced NFZ (e, f, g), where f and g represent reduced NFZ emission spectra after excitation at 230 and 295 nm, respectively. The excitation spectra are presented as solid lines, and emission ones are presented as dashed lines.

Experiments evidenced that the reduced NFZ has a maximum fluorescence at 350 nm (Figure 2). The reaction product showed two excitation wavelengths at 230 and 295 nm. Due to its better selectivity and diminished blank signal, 295 nm was selected as the optimum excitation wavelength.

### 3.2. Optimization of the Reaction Conditions

**Figure 3:**
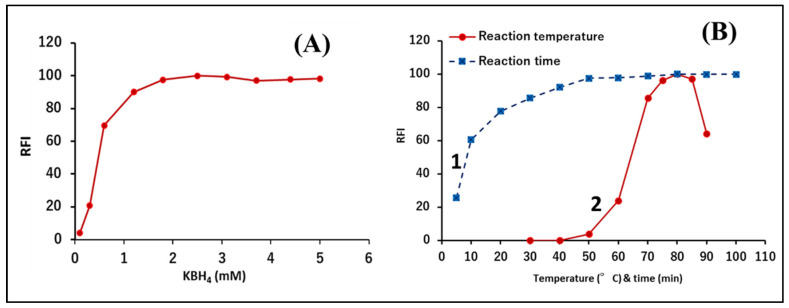
Effect of (**A**) concentration of the reducing agent, potassium borohydride, (**B**) the reaction time (1) and temperature (2) on the relative fluorescence intensity of the reduced NFZ.

Different reaction conditions were studied for the optimization of the analytical procedure. The concentration of the reducing agent was studied using 2.0 mL of potassium borohydride solutions of different concentrations (0.1–5.0 mM). The RFI increased with increasing concentration of potassium borohydride, then a plateau region was reached from 1.8 mM and upward (Figure 3A). We selected 2.5 mM potassium borohydride as the optimum concentration to achieve the best RFI with high robustness.

The study showed that temperature has a substantial effect on the proceeding of the reaction. The reaction was not initiated at temperatures <50 °C, and the maximum RFI was obtained by raising the temperature to 70–85 °C, while the RFI decreased by a further increase in the temperature (Figure 3B); probably due to the decomposition of the fluorescent reaction product at higher temperatures. To ensure good robustness along with the highest RFI, 80 °C was selected to conduct the reaction. The heating time is also another important influencing factor. The study included examination of the heating time effect over intervals from 5 to 100 min. A constant and maximum RFI was reached by heating for 50 min, and it remained constant up to 100 min (Figure 3B). Eventually, heating for 60 min was found to be the most appropriate for the maximum RFI and robustness.

**Figure 4:**
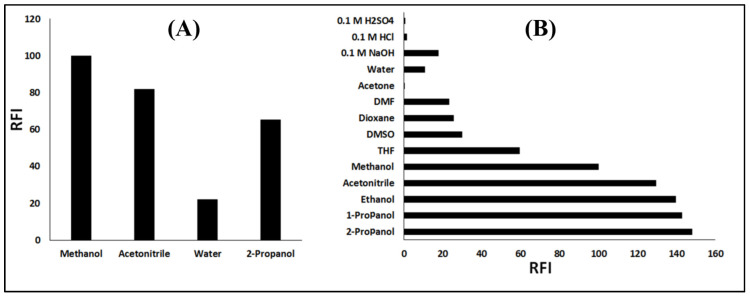
Effect of (**A**) the reaction solvent and (**B**) the diluting solvent on the relative fluorescence intensity of the reduced NFZ.

Another factor that considerably influences the reaction progress and fluorescence of the product is the solvent in which the reducing agent (potassium borohydride) was dissolved. The best RFI intensity was achieved by carrying out the reaction in methanol, while water has a strong inhibitory effect on the reaction, and acetonitrile and 2-propanol gave relatively lower RFI (Figure 4A). This finding agreed with the literature about the reduction of aldehydes and ketones with borohydride that is conventionally carried out in methanol as a solvent [28].

### 3.3. Solvent Dependency of the Reaction Product Fluorescence

Since the diluting solvent in which the RFI is ultimately measured has a profound effect on the fluorescence [19], solvents of different natures have been studied to select the most proper one, including polar protic solvents such as 2-propanol, 1-propanol, methanol, ethanol, and water and polar aprotic solvents such as acetonitrile, tetrahydrofuran (THF), dimethylsulfoxide (DMSO), dimethylformamide (DMF), 1,4-dioxane, and acetone, in addition to acidic and basic aqueous solutions (0.1 M NaOH, 0.1 M HCl, and 0.1 M H_2_SO_4_). The highest RFI was obtained by dilution with 2-propanol, while other solvents either gave poorer RFI or even completely quenched the fluorescence (Figure 4B). Hence, 2-propanol was used as the diluting solvent for the developed method.

The high emission intensity in 2-propanol is most likely related to its high viscosity compared to other tested solvents (viscosity *η* = 2.0 cP at 25 °C) [28,29]. Such high viscosity results in a relatively rigid medium that inhibits the non-radiative deactivation of the lowest excited singlet state and diminishes the collisional loss of energy, giving the highest fluorescence signal. In addition, it is expected that the solvent polarity has a significant effect on the fluorescence intensity [19]. 2-Propanol has intermediate polarity among the tested solvents since its dielectric constant (ε) = 18.3 (25 °C), while the other solvents are either very polar with high ε ≥ 20 (e.g., water, DMSO, DMF, methanol, ethanol, acetonitrile, 1-propanol, and acetone) or weakly polar with low ε such as THF and 1,4-dioxane (ε = 7.6 and 2.21 at 20 °C, respectively) [28,29]. In addition, it is anticipated that intramolecular hydrogen bonding of the phenolic O-H and the nitrogen of the hydrazine side-chain predominates while using 2-propanol as a solvent due to its weaker tendency to H-bonding. Thus, intra-molecular H-bonding leads to the formation of a six-membered ring which assists the co-planarity and rigidity of the molecules and, in turn, favors the fluorescence in 2-propanol (Figure 1B) [23]. In contrast, when the molecules are not involved in intramolecular H-bonding, they have a nonplanar structure (Figure 1C). Thus, it is concluded that 2-propanol positively influences the fluorescence of the reaction product by allied effects, including high viscosity, intermediate polarity [19], and intramolecular H-bonding.

On the other hand, when water was used as a diluting solvent, a strong red-shift of the fluorescence spectra (λ_ex_/λ_em_ of 285/460 nm) with a sharp decrease in the RFI was observed. The high polarity of water favors fast intermolecular H-bonding at the expense of intramolecular H-bonding that predominates in 2-propanol. Intermolecular H-bonding of the lowest excited singlet state (via the proton of the O-H group) with water molecules leads to stabilization of the excited state and decreases the energy difference between the excited and ground states resulting in red-shift and strong decrease in the fluorescence intensity [30]. This behavior is well-known for phenolic compounds [19]. The quenching effect of water on the fluorescence of 1-naphthol compounds is also recognized in the literature [30,31], and it is applicable to the reaction product since it is a substituted 1-naphthol.

The complete quenching of the reaction product fluorescence in acetone may be attributed to two effects. First, hydrogen transfer from the excited 1-naphthol reaction product to the triplet state acetone leading to the formation of a non-fluorescent charge-transfer complex. This phenomenon is known by excited-state proton transfer (ESPR), and it is a specific property of phenols, naphthols, and aromatic amines [32]. The charge transfer complex formation is also a well-known phenomenon that characterizes acetone with a variety of compounds [33,34]. The second effect is the inner filter effect which happens due to the absorption of the excitation or fluorescence light by the quencher. This effect occurs with solvents that show ultraviolet absorption near the excitation or emission wavelength of the analyte [35]. Since acetone has an ultraviolet cut-off of 330 nm [31], it probably quenches the fluorescence of the reaction product by absorption of the excitation light. The inner filter effect is also responsible for the fluorescence quenching in DMF and DMSO due to the absorption of excitation light since their ultraviolet cut-offs are 270 and 265 nm, respectively [29].

### 3.4. Analytical Method Validation for the Standard Analysis

ICH Q2(R1) guidelines [36] were followed during the validation study. The developed method has excellent linearity from 10.0 to 500 ng/mL with a high correlation coefficient (r=0.9999) and small values of standard deviations of residuals, intercept, and slope. The method is highly sensitive for NFZ, with a limit of quantification (LOQ) of 9.0 ng/mL and a limit of detection (LOD) of 2.9 ng/mL. Table 1 shows a summary of the linearity results.

The accuracy of the method was determined by comparison of its results with the results obtained by the reported HPLC method [8] using the Student’s *t*-test and variance ratio *F*-test [37] (Table 2). The calculated values of *t* and *F* were less than the tabulated ones, which proves the absence of significant differences regarding the accuracy and precision of the two methods, respectively.

Intra-day precision was examined by the determination of three different concentrations of NFZ three repetitive times on the same day, while inter-day precision was determined by the determination of three different concentrations of NFZ on three successive days. The results of the precision study are illustrated in Table 3. The small values of standard deviation (SD) and relative standard deviation (%RSD) are evidence of the high precision of the proposed method.

In addition, the effect of small, intended variations in the optimum reaction conditions on the RFI was explored to assess the robustness of the proposed method. No significant change in the RFI of the reaction product by the small variations in potassium borohydride concentration (from 2.0 to 3.0 mM), heating temperature (from 75 to 85 °C), and heating time (from 50 to 70 min). These results confirmed the robustness of the developed method.

### 3.5. Pharmaceutical Application

The developed method was next tested for quality control of NFZ in its tablets. It was applied to determine NFZ in Mediaven tablets containing 5 mg/tablets. The results were compared with that of the reported HPLC method [8] by applying the Student’s t-test and the variance ratio F-test. The calculated t and F values were less than the theoretical values showing that the accuracy and precision of the two methods are not significantly different [20]. The results for the determination of tablets are shown in Table 4.

### 3.6. Application and Validation of the Developed Method for the Analysis of Naftazone in Human Serum

**Figure 5:**
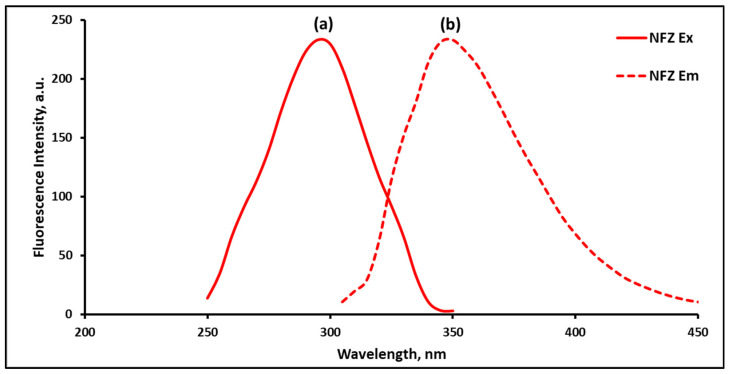
Excitation and emission spectra of NFZ extracted from human serum samples using SALLE upon its reduction with potassium borohyfride, where (a) and (b) represent reduced NFZ excitation and emission spectra using emission setting of 350 nm and excitation setting of 295 nm, respectively.

The developed method represents the first fluorometric analysis of naftazone, and hence it was characterized by its high sensitivity and selectivity. The level of naftazone in serum samples ranges from 50 to 200 ng/mL [7], which lies in the linear range of the developed method. Thus, we intended to apply the developed method for the determination of naftazone in human serum. In order to extract naftazone in an easy and time-saving way, the simple and efficient SALLE [38,39,40] was adopted for the first time for naftazone. The serum samples were mixed with acetonitrile then phase separation was induced through salting out using NaCl. The separated acetonitrile phase, which contains naftazone, is then reacted with potassium borohydride to yield the fluorescent product. At first, serum samples were spiked with different naftazone concentrations to construct the calibration curve, and the method showed excellent linearity in the range of 10–500 ng/mL following the regression equation of Y = 1.12X + 10.2. The LOD and LOQ were found to be 3.6 and 10 ng/mL, where the S/N ratio at LOD was 3 and at LOQ was more than 5, and the accuracy and precision at LOQ were acceptable (<20%, Table 5), which is in accordance with the FDA guidelines [17]. The fluorescence spectra of naftazone (200 ng/mL) extricated from human serum samples is shown in Figure 5. The accuracy and precision of the developed method were studied across the linear range according to the procedure mentioned earlier in the experimental section, and the results are abridged in Table 5. The method showed good accuracy (relative error ranged from −10.7 to 11.8%) and acceptable precision (RSD ≤ 12.5%). Then, the recovery of naftazone from plasma using SALLE was calculated following the procedure in the experimental section, and the results are cited in Table 6. The method showed good recovery in the range of 92.3–106.5 with excellent recovery precision (RSD ≤ 6.8%). All these results demonstrate the reliability and applicability of the developed method for the determination of naftazone in human serum samples for therapeutic monitoring purposes.

### 3.7. Results of the Kinetic Study of NFZ Reaction with Potassium Borohydride

**Figure 6:**
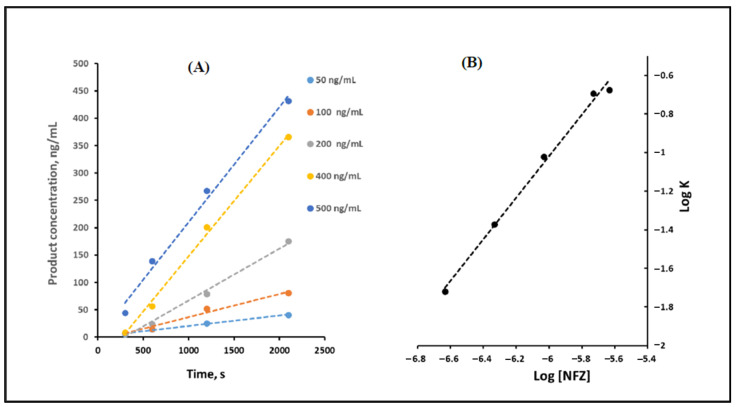
(**A**) Product concentration (ng/mL) versus time (seconds) curve for increasing concentrations of NFZ, and (**B**) log K versus log [NFZ] curve.

A kinetic study of the reaction of NFZ and potassium borohydride was performed for a better understanding of the reaction mechanism [18]. Figure 6A shows the product concentration (ng/mL)–time (seconds) curves for different concentrations of NFZ under the optimum experimental conditions. The initial reaction rates (K) at each concentration were calculated from the slopes of these straight lines.

In order to determine the order of the reaction, the graphical method was adopted by plotting log K versus log [NFZ]. The graph is a straight line whose correlation coefficient r = 0.997 and its slope = 1.08 (Figure 6B). From Equation (2), the slope of this line expresses the order of the reaction. Since potassium borohydride exists in higher excess than NFZ under the optimum experimental conditions, the reaction is considered pseudo-first-order.

**Figure 7:**
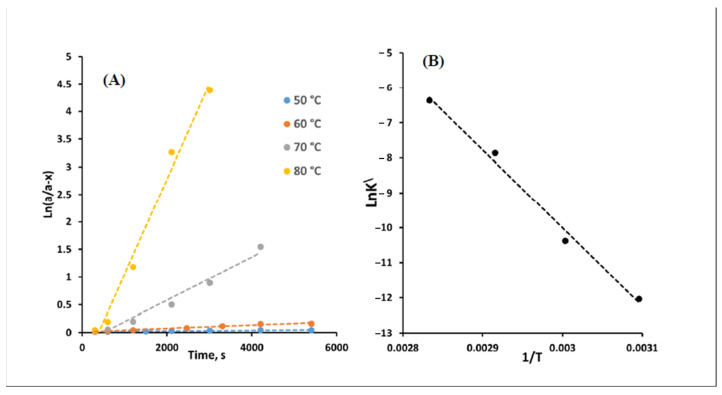
(**A**) Semi logarithmic plot for the first-order reaction of NFZ and potassium borohydride and (**B**) Arrhenius plot for the reaction of NFZ and potassium borohydride.

Therefore, plots of ln (a/a-x) versus time (seconds) were graphed according to the integrated first-order rate law (Equation (3)), as seen in Figure 7A. The rate constants K’ were calculated from the slopes of the obtained straight lines and found to be 5.96 × 10^−6^, 3.10 × 10^−5^, 3.88 × 10^−4^, 1.72 × 10^−3^ s^−1^ at 50, 60, 70, and 80 °C, respectively. Then Arrhenius plot (log K versus 1/T) was graphed as realized in Figure 7B, and the Arrhenius equation was derived as follows:

**Ln K’ = 56.8 − 22,257.1/T** (5)

From the slope, the activation energy of the reaction was calculated and found to be 43.8 kcal/mol. This value refers to the minimum amount of energy needed to convert the reactant molecules into a product. The calculated value agreed well with the reported value for reduction reactions involving borohydride [41].

### 3.8. Assessment of Method Greenness

In the 1990s, green chemistry has been evolved to avoid harm and toxicity to humans and the environment. Indeed, analytical chemistry is considered a major active area of green chemistry research [42]. Hence, it has been a matter of interest for our research team to contribute to the idea of sustainable development, resulting in many publications in this area [43,44,45,46,47]. Thus, in this work, we tried to conserve the principles of green analytical chemistry (GAC) and, in the meantime, provide the best analytical performance parameters to the developed method. Herein, we assess the developed method on the bases of three analytical metrics: the NEMI labeling, the analytical eco-scale [16], and the analytical greenness calculator (AGREE) [48] tools.

**Figure 8:**
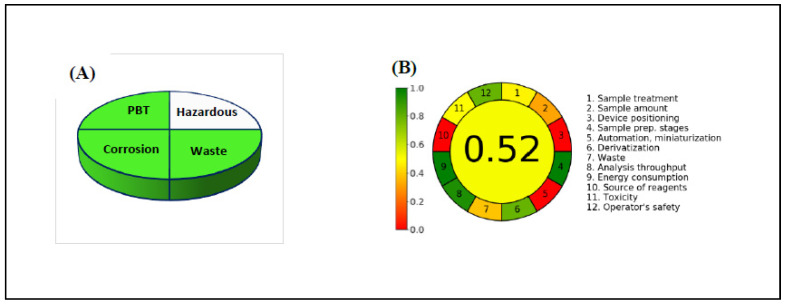
Results of (**A**) NEMI and (**B**) AGREE analysis for the developed method.

The NEMI labeling approach depends on a pictographic illustration divided into four parts; each one is colored green when one of the following criteria is met: the reagents used are not in the persistent, toxic, and bio-accumulative (PBT) list [49], the waste generated is not listed in the hazardous waste (HW) list [50], the pH of the sample is within the range of 2–12 (non-corrosive), and the volume of the waste generated during the analysis of a sample does not exceed 50 mL. We found that the proposed method fulfilled these criteria except for the second one as shown in Figure 8A, where methanol and acetonitrile are listed in the HW list due to ignitability [50]. However, most solvent selection guides classified methanol as a “recommended” solvent [51]. Additionally, methanol and acetonitrile are used in the developed procedure for the preparation of the standard solution and extracts of tablets and serum only, and the volume taken for final analysis is small (≤1 mL for a sample).

The analytical eco-scale metric was also applied to evaluate the greenness of the method. Calculation of the analytical eco-scale is based on giving penalty points (PPs) for each parameter of the analytical procedure (reagent hazard, amount of reagent, waste, energy consumption, occupational hazards), where the ideal analytical method has an analytical eco-scale score of 100. The proposed method scored 76 (Table 7), which means that it is an excellent green analytical method [52].

The analytical greenness calculator, AGREE [49], was also utilized as a more comprehensive tool for the evaluation of the greenness of the proposed method through examination of several criteria, including sample amount, treatment, throughput, waste, reagent source, and safety, and energy consumption. The result of the assessment of the proposed method illustrated in Figure 8B revealed its greenness.

In sum, the developed method is promising for widespread application in quality control laboratories without strong harmful influences on the environment or the operators.

### 3.9. Comparison of the Developed Method with the Published Methods for NFZ

Eventually, it is important to compare the performance of the developed method with that of methods reported in the literature (Table 8). Concerning sensitivity, the proposed method is 25–100 times more sensitive than the reported spectrophotometric methods for NFZ [3]. Additionally, it is about four times more sensitive than the polarographic and TLC methods for NFZ [5,7] and 11 times more sensitive than the reported HPLC method [7]. Despite the cathodic stripping voltammetric method [4] and the LC-MS/MS methods having relatively superior sensitivity to the proposed method, they have some restrictions due to the limited availability of the instruments in quality control laboratories. In addition, the exposure to toxic mercury in the case of voltammetry [4] and the instrumental complexity and high costs in the case of LC-MS/MS [9] limit the widespread applications of these methods. On the contrary, the developed method employed simple and available reagents and instruments, and the analytical procedure is obviously feasible. These merits made the developed method a good choice for quality control of NFZ.

## 4. Conclusions

The first spectrofluorimetric method for NFZ was designed and validated based on the reduction-triggered fluorogenic derivatization with potassium borohydride. The method is highly sensitive, with an LOD of 2.9 ng/mL. The greenness of the developed method was confirmed by applying three evaluation metrics. This merit, with the availability and low cost of the reagent used, in addition to the accuracy, precision, and sensitivity of the proposed method, made it an excellent substitute to existing methods for the analysis of NFZ in quality control laboratories. Commercially available tablets containing NFZ were successfully analyzed by the developed method with a high %recovery (100.33 ± 1.48). Furthermore, the method was applied successfully for the determination of NFZ in human serum samples using SALLE for NFZ for the first time.

## Figures and Tables

**Table 1:** Analytical parameters for determination of NFZ by the proposed fluorimetric method.

Parameter	Value
λ_excitation_/λ_emmision_ (nm)	295/350
Range (ng/mL)	10.0–500
Limit of detection (LOD) (ng/mL)	2.9
Limit of quantification (LOQ) (ng/mL)	9.0
r	0.9995
Slope (b)	1.15
Intercept (a)	4.75
Residuals Standard deviation (S_y/x_)	2.30
Intercept standard deviation (S_a_)	6.97
Slope standard deviation (S_b_)	0.01
%RSD	0.67
%Error	0.46

LOD = 3.3 S_a_/_b_. LOQ = 10 S_a_/_b_.

**Table 2:** Application of the presented spectrofluorimetric method for NFZ determination in pure form and statistical comparison with the obtained results adopting the reported HPLC method.

Parameters	Proposed Method			Comparison Method [8]	
	Conc. Taken (ng/mL)	Conc. Found (µg/mL)	% Found ^a^	Conc. Taken (ng/mL)	% Found ^a^
	10	9.934	99.3	500	100.0
	50	50.386	100.8	1000	100.8
	100	101.87	101.9	2000	99.7
	200	198.008	99.0		
	400	397.115	99.3		
	500	502.710	100.5		
**Mean ± SD**			100.1 ± 1.1		100.2±0.5
***t***			0.05 (2.36) ^b^		
***F***			4.36 (19.30) ^b^		

^a^ Each result is three determinations average. ^b^ Tabulated *t* and *F* values are presented between parentheses, where *p* = 0.05 [37].

**Table 3:** Results of the accuracy and precision study of the proposed spectrofluorimetric method for NFZ analysis.

Parameter	Intra-Day Precision			Inter-Day Precision		
	Conc. (ng/mL)			Conc. (ng/mL)		
	50	200	500	50	200	500
**% Found**	98.7	101.1	99.2	100.4	100.3	99.4
	100.8	99.3	100.4	100.8	100.6	99.0
	101.8	100.6	98.4	98.7	101.9	101.3
**Mean**	100.4	100.3	99.4	100.0	100.9	99.9
**% RSD**	1.6	1.0	1.0	1.1	0.8	1.2
**% Error**	0.9	0.6	0.6	0.7	0.5	0.7

**Table 4:** Application of the proposed spectrofluorimetric method for the determination of NFZ in tablet and statistical comparison of the data with those obtained by the reported HPLC method.

Parameters	Proposed Method			Comparison Method [8]	
	Conc. Taken (ng/mL)	Conc. Found (ng/mL)	% Found ^a^	Conc. Taken (ng/mL)	% Found ^a^
	50	50.91	101.82	500	99.8
	200	200.64	100.32	1000	98.9
	500	494.3	98.86	2000	100.4
**Mean ± SD**			100.33 ± 1.48		99.73 ± 0.80
***t***			0.62 (2.78) ^b^		
***F***			3.41 (19.00) ^b^		

^a^ Each result is t is three determinations average. ^b^ Values between parentheses are the tabulated *t* and *F* values, respectively, at *p* = 0.05 [37].

**Table 5:** Accuracy and precision data for of naftazone determination in spiked serum samples.

Spiked Level (ng/mL) (n = 3)	Accuracy (% Found)	Precision RSD (%)
10 (LOQ)	11.8	12.5
50	−10.7	2.6
100	1.7	1.6
200	−3.3	7.2
500	0.6	0.6

**Table 6:** The recovery of naftazone from spiked serum samples using SALLE.

Spiked Level (ng/mL)	Recovery (% Found)	Precision RSD (%)
10 (LOQ)	99.4	6.8
50	92.3	2.2
100	94.9	3.0
200	106.5	3.8
500	99.3	1.0

**Table 7:** Penalty points for the proposed method and calculation of its analytical eco-scale score.

Reagent (Volume, mL)	Number of Pictograms	Signal Word	Penalty Points
Potassium borohydride	3	Danger	6
Acetonitrile	2	Danger	4
2-Propanol	2	Danger	4
Methanol	3	Danger	6
**Instruments**			
Spectrofluorimetry			0
Heater			2
Occupational hazard			0
Waste			2
**Penalty points**			Σ24
**Score**			76 (excellent green analysis)

**Table 8:** Comparison of the proposed method’s performance with that of the literature methods.

Method	Conditions	Sensitivity (LOD) (ng/mL)	Ref.
Spectrophotometry	Colorimetric measurement in 2 mM NaOH at 494 nm Complexation with Cu (II) at pH 7.6 and Colorimetric measurement at 512 nm Complexation with Ni (II) at pH 7.6 and Colorimetric measurement at 506 nm Complexation with Co (II) at pH 8.0 and Colorimetric measurement at 498 nm Complexation with Zn (II) at pH 7.6 and Colorimetric measurement at 502 nm	70 80 210 450 270	[3]
Cathodic stripping voltammetry	0.05 M NaOH	1.1	[4]
Polarography	pH 7.0	11	[5]
Differential pulse polarography	Complexation with Cu(II) at pH 5.0 or 7.4	N/A	[6]
TLC	Spraying the TLC plates sprayed with lead acetate followed by simultaneous reflectance and transmittance measurements at 520 nm	10	[7]
HPLC-UV detection	Methanol:0.02 M NaH_2_PO_4_ (60:40, *v*/*v*) with pH 6.0 as a mobile phase and phenyl column.	32	[8]
LC-MS/MS	Gradient elution of two mobile phases: 0.1% formic acid-10 mM ammonium formate (A) and acetonitrile (B) and C18 column	0.1	[9]
Spectrofluorometry	Potassium borohydride/80 °C/60 min	2.8	Present method

## Data Availability

Data are available upon request.

## References

[1] Sweetman S.C. (2009). Martindale: The Complete Drug Reference.

[2] Acton Q.A. (2013). Parkinson’s Disease: New Insights for the Healthcare Professional.

[3] Ibrahim F.A., El-Enany N.M., El-Shaheny R.N., Mikhail I.E. (2015). Spectroscopic Studies on Naftazone and Its Metal Complexes with Analytical Applications for Quality Control of Tablets. Anal. Methods.

[4] Khodari M., Vire J.C., Patriarchs G.J., Ghandour M.A. (1990). Cathodic and Adsorptive Stripping Votammetry of Naftazone. Anal. Lett..

[5] Viré J.-C., Patriarche G.J., Christian G.D. (1979). Electrochemical Study of 1,2-Naphthoquinone-4-Sulphonate and 1,2-Naphthoquinone Semicarbazone. Fresenius Z. Anal. Chem..

[6] Maali N.A., Vire J.-C., Patriarche G.J., Ghandour M.A. (1990). Polarographic Study of Copper (II) Complexes with Some Naphthoquinonic Derivatives. Anal. Lett..

[7] Bressolle F., Bres J., Brun S., Rechencq E. (1979). Quantitative determination of drugs by in situ spectrophotometry of chromatograms for pharmacokinetic studies. I. Sulpiride and other benzamides, vincamine, naftazone (author’s transl). J. Chromatogr..

[8] Walash M.I., Belal F., El-Enany N., Eid M., El-Shaheny R.N. (2011). Stability-Indicating HPLC Method for Determination of Naftazone in Tablets. Application to Degradation Kinetics and Content Uniformity Testing. J. Chromatogr. Sci..

[9] Park J.-A., Abd El-Aty A.M., Zheng W., Kim S.-K., Cho S.-H., Choi J.-M., Hacimuftuoglu A., Jeong J.H., Wang J., Shim J.-H. (2018). Simultaneous Determination of Clanobutin, Dichlorvos, and Naftazone in Pork, Beef, Chicken, Milk, and Egg Using Liquid Chromatography-Tandem Mass Spectrometry. Food Chem..

[10] El-Shaheny R., Al-Khateeb L.A., El Hamd M.A., El-Maghrabey M. (2021). Correction Pen as a Hydrophobic/Lipophobic Barrier Plotter Integrated with Paper-Based Chips and a Mini UV-Torch to Implement All-in-One Device for Determination of Carbazochrome. Anal. Chim. Acta.

[11] Mantoanelli J.O.F., Gonçalves L.M., Pereira E.A. (2020). Dansyl Chloride as a Derivatizing Agent for the Analysis of Biogenic Amines by CZE-UV. Chromatographia.

[12] Walash M.I., Belal F., El-Enany N., El-Maghrabey M.H. (2011). Simple and Sensitive Spectrofluorimetric Method for the Determination of Pregabalin in Capsules through Derivatization with Fluorescamine. Luminescence.

[13] Ibrahim F., El-Enany N., El-Shaheny R.N., Mikhail I.E. (2015). Validated Spectrofluorimetric and Spectrophotometric Methods for the Determination of Brimonidine Tartrate in Ophthalmic Solutions via Derivatization with NBD-Cl. Application to Stability Study. Luminescence.

[14] Walash M.I., Belal F., El-Enany N., El-Maghrabey M.H. (2012). Spectrofluorimetric Determination of Oseltamivir Phosphate through Derivatization with O-Phthalaldehyde. Application to Pharmaceutical Preparations with a Preliminary Study on Spiked Plasma Samples. Luminescence.

[15] El-Maghrabey M., Suzuki H., Kishikawa N., Kuroda N. (2021). A Sensitive Chemiluminescence Detection Approach for Determination of 2,4-Dinitrophenylhydrazine Derivatized Aldehydes Using Online UV Irradiation–Luminol CL Reaction. Application to the HPLC Analysis of Aldehydes in Oil Samples. Talanta.

[16] Tobiszewski M., Marć M., Gałuszka A., Namiesnik J. (2015). Green Chemistry Metrics with Special Reference to Green Analytical Chemistry. Molecules.

[17] U.S. Department of Health and Human Services-Food and Drug Administration Bioanalytical Method Validation Guidance for Industry. https://www.fda.gov/downloads/drugs/guidances/ucm070107.Pdf.

[18] Upadhyay S.K. (2006). Chemical Kinetics and Reaction Dynamics.

[19] Ohannesian L., Streeter A.J. (2002). Handbook of Pharmaceutical Analysis.

[20] Mahmoud A.M., Khalil N.Y., Darwish I.A., Aboul-Fadl T. (2009). Selective Spectrophotometric and Spectrofluorometric Methods for the Determination of Amantadine Hydrochloride in Capsules and Plasma via Derivatization with 1,2-Naphthoquinone-4-Sulphonate. Int. J. Anal. Chem..

[21] Kalpana G.L., Ravisankar P., Rao G.D., Raju M.B. (2016). A Novel Spectrofluorimetric Method for the Determination of Amisulpride in Bulk and Pharmaceutical Formulation. Der Pharm. Lett..

[22] Ma J., Del Vecchio R., Golanoski K.S., Boyle E.S., Blough N.V. (2010). Optical Properties of Humic Substances and CDOM: Effects of Borohydride Reduction. Environ. Sci. Technol..

[23] Buna M., Aaron J.-J., Prognon P., Mahuzier G. (1996). Effects of PH and Solvent on the Fluorescence Properties of Biomedically Important Benzamides. Application to Determination in Drugs and in Human Urine. Analyst.

[24] Xu Y., Wei Y. (2010). CeCl_3_-Catalyzed Reduction of Methyl Esters of Carboxylic Acids to Corresponding Alcohols with Sodium Borohydride. Synth. Commun..

[25] Kuehne M.E., Shannon P.J. (1977). Reduction of Amides and Lactams to Amines by Reactions with Phosphorus Oxychloride and Sodium Borohydride. J. Org. Chem..

[26] Caddick S., Judd D.B., Lewis A.K.K., Reich M.T., Williams M.R. (2003). A Generic Approach for the Catalytic Reduction of Nitriles. Tetrahedron.

[27] Ward D.E., Rhee C.K. (1988). Chemoselective Reductions with Sodium Borohydride. Aldehydes vs. Ketones. Synth. Commun..

[28] Isac-García J., Dobado J.A., Calvo-Flores F.G., Martínez-García H. (2016). Green Chemistry Experiments. Experimental Organic Chemistry: Laboratory Manual.

[29] Smallwood I.M. (1996). Handbook of Organic Solvent Properties.

[30] Henson R.M.C., Wyatt P.A.H. (1975). Fluorescence and Flash Photolysis Comparison of 1-Hydroxynaphthalene-2-Sulphonate and 1-Hydroxynaphthalene-4-Sulphonate Ions in Aqueous Solutions of Various Acidities. J. Chem. Soc. Faraday Trans..

[31] Agmon N., Huppert D., Masad A., Pines E. (1991). Excited-State Proton Transfer to Methanol-Water Mixtures. J. Phys. Chem..

[32] Pappayee N., Mishra A.K. (2000). Excited State Proton Transfer of Some Substituted Naphthols in Liposomes. Indian J. Chem..

[33] Bryce-Smith D., Gilbert A., Cox A., Bryce-Smith D., Gilbert A. (1989). Photo-Reduction and -Oxidation. Photochemistry.

[34] Loutfy R.O., Yip R.W., Dogra S.K. (1977). The Triplet State of Ketones in Solution: Quenching of Triplet Acetone by Olefins. Tetrahedron Lett..

[35] Williams R.T., Bridges J.W. (1964). Fluorescence of Solutions: A Review. J. Clin. Pathol..

[36] ICH Expert Working Group Validation of Analytical Procedures: Text and Methodology, Q2(R1). Proceedings of the International Conference on Harmoanization.

[37] Miller J.N., Miller J.C. (2010). Statistics and Chemometrics for Analytical Chemistry.

[38] Zhang J., Wu H., Kim E., El-Shourbagy T.A. (2009). Salting-out Assisted Liquid/Liquid Extraction with Acetonitrile: A New High Throughput Sample Preparation Technique for Good Laboratory Practice Bioanalysis Using Liquid Chromatography-Mass Spectrometry. Biomed. Chromatogr..

[39] El-Maghrabey M., Kishikawa N., Kuroda N. (2016). 9,10-Phenanthrenequinone as a Mass-Tagging Reagent for Ultra-Sensitive Liquid Chromatography-Tandem Mass Spectrometry Assay of Aliphatic Aldehydes in Human Serum. J. Chromatogr. A.

[40] El-Maghrabey M., Kishikawa N., Kuroda N. (2018). Novel Isotope-Coded Derivatization Method for Aldehydes Using 14 N/15 N-Ammonium Acetate and 9,10-Phenanthrenequinone. Anal. Chem..

[41] Iben Ayad A., Luart D., Ould Dris A., Guénin E. (2020). Kinetic Analysis of 4-Nitrophenol Reduction by “Water-Soluble” Palladium Nanoparticles. Nanomaterials.

[42] Anastas P.T. (1999). Green Chemistry and the Role of Analytical Methodology Development. Crit. Rev. Anal. Chem..

[43] El-Shaheny R.N., El-Maghrabey M.H., Belal F.F. (2015). Micellar Liquid Chromatography from Green Analysis Perspective. Open Chem..

[44] El-Shaheny R.N., El-Enany N.M., Belal F.F. (2014). A Green HPLC Method for the Analysis and Stability Study of Flavoxate HCl Using Micellar Eluent. Anal. Methods.

[45] El-Shaheny R.N. (2015). Stability-Indicating Micellar LC Methods with Time-Programmed UV Detection for Determination of Three Oxicams in Pharmaceuticals with Direct Injection of Gel and Suppositories. J. Liq. Chromatogr. Relat. Technol..

[46] Ibrahim F.A., El-Enany N., El-Shaheny R.N., Mikhail I.E. (2015). Simultaneous Determination of Desloratadine and Montelukast Sodium Using Second-Derivative Synchronous Fluorescence Spectrometry Enhanced by an Organized Medium with Applications to Tablets and Human Plasma. Luminescence.

[47] Ahmed A.B., Gamal M., Naguib I.A., Ali H.M., Abdallah F.F. (2022). Environmental Impact of the Reported Chromatographic Methods for the Determination of the First FDA-Approved Therapy for COVID-19 Patients, Remdesivir: A Comparative Study. Microchem. J..

[48] Pena-Pereira F., Wojnowski W., Tobiszewski M. (2020). AGREE—Analytical Greenness Metric Approach and Software. Anal. Chem..

[49] United States Environmental Protection Agency, Persistent Bioaccumulative Toxic (PBT) Chemicals Covered by the TRI Program. https://www.epa.gov/toxics-release-inventory-tri-program/persistent-bioaccumulative-toxic-pbt-chemicals-covered-tri.

[50] United States Environmental Protection Agency Hazardous Waste Listings, First Update. https://www.epa.gov/sites/production/files/2016-01/documents/hw_listref_sep2012.pdf.

[51] Tobiszewski M. (2016). Metrics for Green Analytical Chemistry. Anal. Methods.

[52] Gałuszka A., Migaszewski Z.M., Konieczka P., Namieśnik J. (2012). Analytical Eco-Scale for Assessing the Greenness of Analytical Procedures. Trends Anal. Chem..

